# Study the charging process of moving quantum batteries inside cavity

**DOI:** 10.1038/s41598-023-37800-y

**Published:** 2023-07-01

**Authors:** Maryam Hadipour, Soroush Haseli, Hazhir Dolatkhah, Maryam Rashidi

**Affiliations:** 1 Faculty of Physics, Urmia University of Technology, Urmia, Iran; 2 School of Physics, Institute for Research in Fundamental Sciences (IPM), P.O. Box 19395-5531 Tehran, Iran; 3 RCQI, Institute of physics, Slovak Academy of Sciences, Dúbravská Cesta 9, 84511 Bratislava, Slovakia; 4 Department of Medical Physics, Faculty of Medicine, Urmia University of Medical Sciences, Urmia, Iran

**Keywords:** Physics, Quantum physics, Quantum information, Quantum mechanics, Qubits

## Abstract

In quantum mechanics, quantum batteries are devices that can store energy by utilizing the principles of quantum mechanics. While quantum batteries has been investigated largely theoretical, recent research indicates that it may be possible to implement such a device using existing technologies. The environment plays an important role in the charging of quantum batteries. If a strong coupling exists between the environment and the battery, then battery can be charged properly. It has also been demonstrated that quantum battery can be charged even in weak coupling regime just by choosing a suitable initial state for battery and charger. In this study, we investigate the charging process of open quantum batteries mediated by a common dissipative environment. We will consider a wireless-like charging scenario, where there is no external power and direct interaction between charger and battery. Moreover, we consider the case in which the battery and charger move inside the environment with a particular speed. Our results demonstrate that the movement of the quantum battery inside the environment has a negative effect on the performance of the quantum batteries during the charging process. It is also shown that the non-Markovian environment has a positive effect on improving battery performance.

## Introduction

There exist a thermodynamic role for batteries, which is that of a work reservoir. It is well known that traditional batteries are electrochemical devices that store energy from external sources and provide that energy to other machines, allowing them to be operated remotely without the need for a power source. In recent years, batteries have become essential devices, in both size and storage capacity, ranging from large car traction batteries of 500kWh to tiny 100mWh cells used in small electronic devices^1^. With the constant miniaturization of such devices, batteries are also becoming smaller and smaller, so as their unit cells approach molecules and atoms, quantum mechanical effects must be taken into consideration when describing them^2–5^. In quantum mechanics, quantum battery (QB) is a d-dimensional energy storage quantum system with non-degenerate energy levels that relies on the principles of quantum mechanics to operate and store energy^6,7^. Despite the fact that QBs are still a theoretical concept, some progress has been made in developing experimental systems that demonstrate some of the principles involved in quantum energy storage^8–12^. QBs are designed using quantum theory concepts such as quantum coherence and quantum entanglement. In addition to being more efficient and having a greater energy density, QBs have the potential to be smaller and lighter than conventional classical batteries. What are the best conditions for a QB? That is the first question we should answer when discussing about QBs. It should be noted that one of the most important characteristics of a good QB is its ability to store as much energy as possible in the shortest possible time. It is also important for a QB to be able to discharge the energy sufficiently in an optimal period of time. The internal energy of a QB, as well as the work that can be extracted from it, are important indicators of the battery’s quality^13–20^.

Quantum mechanics traditionally deals with isolated systems that are completely isolated from their surrounding environment. The concept of open quantum systems arises from the interaction between physical systems and their surroundings. As a result of these interactions, energy, information, or particles may be exchanged between the system and its environment. Decoherence is one of the most challenging aspects of studying open quantum systems. In quantum physics, decoherence occurs when interaction of the quantum system with the environment lead to a loss of quantum coherence^21–23^.

Since environmental effects on quantum systems cannot be avoided, studying QBs from the open quantum systems perspective seems essential^24–30^. A wide range of research has previously been conducted on the effects of environmental parameters and memory effects on the charging and discharging processes of QBs^25–30^. As we will model the QB using open quantum system theory, we will first briefly discuss about the open quantum systems. It is possible to classified the evolution of open quantum systems into two categories: Markovian and non-Markovian evolution^21–23^. In Markovian evolution, the future state of a system depends only on its current state, not on its past. Consequently, the evolution of the system has no memory and can be modeled using a Lindblad master equation (differential equation which describes the evolution of the system’s density matrix over time). In other words, in Markovian regime, the state of the system at time $t+1$ depends on the state of the system at time *t* alone and not on the state of the system at earlier times. Mathematically, this can be expressed as $\rho _{t+1}=\Phi (\rho _t)$, where $\rho _t$ is the density matrix of the system at time *t*, and $\Phi$ is the quantum dynamical map of the system’s evolution. In systems with weak couplings to their environments, or in those with short-range interactions, Markovian properties can emerge. In contrast, non-Markovian evolution involves a system whose future state is dependent on both its current and past states. Accordingly, we can say that memory plays a crucial role in the evolution of a system. Non-Markovian evolution means that the state of an open quantum system at time $t+1$ depends not only on its state at time *t*, but also on its state at every previous time. Mathematically, this can be expressed as $\rho _{t+1}=\Phi (\rho _t,\rho _{t-1},\rho _{t-2},...)$. In non-Markovian evolution, the system may exhibit delayed or oscillatory responses to external perturbations due to memory effects. It should be noted that evolution that does not follow the Markovian model arises when a system is strongly coupled with its environment or when there is long-range interaction between the system and the environment. There are several implications of non-Markovian evolution for the dynamics of quantum systems, including quantum revivals, decoherence-free subspaces, and quantum memory effects. In this study, the charging process of open QBs will be studied in both a Markovian and a non-Markovian regime. While the strong coupling regime is ideal for the highest battery charging efficiency, the weak coupling regime will also be investigated here. By selecting the appropriate initial state for the battery and the charger, QB can be charged to an acceptable level in weak coupling regime, i.e. when Markovian evolution occurs^31^. In recent works, the charging process has been improved by using an intermediate quantum object between the charger and the battery^31,32^.

In the present study, we are focusing on the strategy presented in Ref.^31^. As well as charger-mediated energy transfer, Tabesh et al. have considered an environment-mediated case for the open QBs in Ref.^31^. The scenario includes a realistic scenario for spontaneous discharge of QBs and energy leakage from the batteries. In their model the composite system, including the QB and charger, is analyzed as a two-qubit system. They also suggest that the QB can be charged by using the environment as an intermediary, without an external field or direct connection to the charger. A scenario in which this type of charging process could occur is known as a wireless-like charging process. We consider the case in which the system, including QB and charger, moves within the environment with particular speed. In this study, it will be investigated how the moving of the QB and charger affects the charging process of QB. The moving of quantum systems has been shown to have a negative effect on the charging process and can lead to a reduction in the charging performance of a QB. This work is organized in the following manner: In Sec. II, the short introduction about the amount of work that one can extract from a QB has been provided. In Sec. III, the model of charging process will be introduced. In Sec. IV the details of the charging process of the QB will be studied. The results will be summarized in Sec. V.

## Ergotropy

Let us start from a process that the QB is thermally isolated and does not undergo any heat exchange with its surroundings. Furthermore, the process is cyclic, i.e., at the end of process the system returns to its initial Hamiltonian. Any such process can be given by a unitary transformation,

1 $$\begin{aligned} U(t)=\exp \left\{ -i \int _0^t d t^{\prime }[H_B+V(t^{\prime })]\right\} , \end{aligned}$$

in which $H_B$ is the Hamiltonian of the QB and $V(t^{\prime })$ is the time dependent fields that will be used to extract energy from the QB and constant $\hbar$ has been set equal to 1 ($\hbar =1$) throughout the paper. Since the process is cyclic, $V(t^{\prime })$ vanishes at the beginning and at the end, $V(0) = V(t) = 0$. The work extracted by such a procedure is

2 $$\begin{aligned} W(\rho _B)= Tr(H_B\rho _B) -Tr(H_BU \rho _B U^{\dagger }), \end{aligned}$$

where $\rho _B$ is the state of QB. By a proper choice of $V(t^{\prime })$, any unitary transformation *U* can be generated. Therefore, the maximal amount of work that one can extract from a QB in a cyclic unitary process, known as ergotropy^19^, is given by

3 $$\begin{aligned} {\mathscr {W}}= Tr(H_B\rho _B) -\min _UTr(H_BU \rho _B U^{\dagger }), \end{aligned}$$

where the minimum is taken over the set of all accessible unitary transformations. It has been shown that for any given state $\rho _B$ there is a unique state that maximizes the above relation, this state is called the passive state^33–36^ and one can obtain this state via some unitary transformation that rearranges the eigenvalues of $\rho _B$ in non-increasing order. In other words, the maximal extractable work can be written as^37–39^

4 $$\begin{aligned} {\mathscr {W}}= Tr(H_B\rho _B) -Tr(H_B\sigma _{B}), \end{aligned}$$

where $\sigma _{B}$ is passive state of $\rho _B$. The passive state $\sigma _{B}$ has a non-increasing population with respect to its Hamiltonian $H_B$ and $[H_B,\sigma _{B}] = 0.$ Furthermore, if one can write the spectral decomposition of the density matrix of QB and its corresponding Hamiltonian in the following way

5 $$\begin{aligned} \rho _B= & {} \sum _{i=1}^{d} p_i \vert p_i \rangle \langle p_i \vert \quad p_1 \ge p_2 \ge ... \ge p_d, \end{aligned}$$

6 $$\begin{aligned} H_B= & {} \sum _{i=1}^{d} \varepsilon _i \vert \varepsilon _i \rangle \langle \epsilon _i \vert \quad \varepsilon _1 \le \varepsilon _2 \le ... \le \varepsilon _d, \end{aligned}$$

where *d* is the dimension of the Hilbert space and $p_i$ and $\epsilon _i$ are the eigenvalues of the density matrix $\rho _B$ and Hamiltonian $H_B$ respectively. $\vert p_i \rangle$ and $\vert \epsilon _i \rangle$ are the eigenstates of the density matrix $\rho _B$ and Hamiltonian $H_B$ respectively. So, the passive state $\sigma _B$ can be written as^35^

7 $$\begin{aligned} \sigma _B=U \rho _B U^{\dagger }=\sum _{i=1}^{d} p_i \vert \epsilon _i \rangle \langle \epsilon _i \vert , \end{aligned}$$

and the ergotropy can be obtained as^19^

8 $$\begin{aligned} {\mathscr {W}}=\sum _{i,j}^d p_i \epsilon _j\left( \left| \left\langle p_i \mid \epsilon _j\right\rangle \right| ^2-\delta _{i, j}\right) , \end{aligned}$$

where $\delta _{i, j}$ is the Kronecker delta function.

## Scenario of charging process

**Figure 1:**
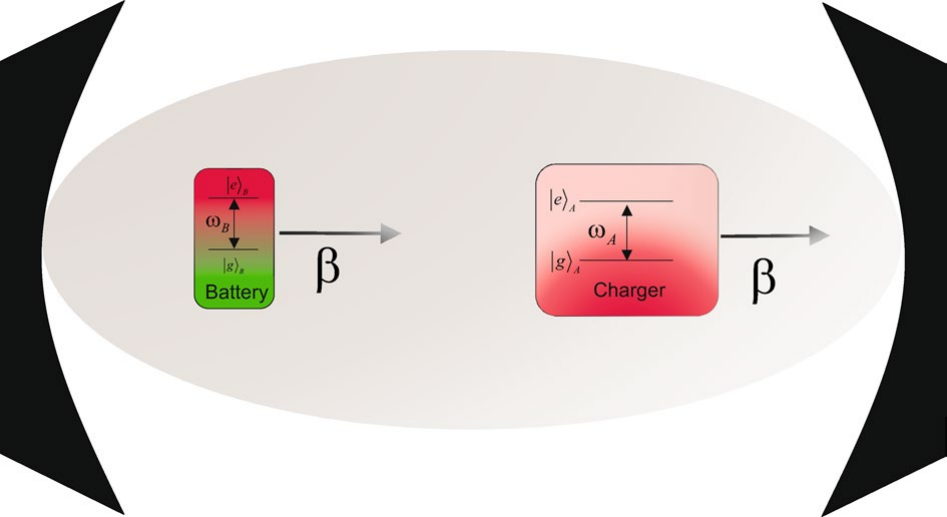
Schematic representation of the model.

The model consists of three subsystems: a quantum charger *A*, a QB *B*, and a common environment *E* that is acting as a mediator between QB and charger. A schematic representation of the model is shown in Fig. 1.

In this scenario, the QB and charger are both considered as the two-level systems with excitation state $\vert e_j \rangle$ and ground state $\vert g_j \rangle$, with two qubits having the same transition frequency $\omega _1=\omega _2$. Here, the first qubit ($j=1$) with transition frequency $\omega _1=\omega _A$ will be considered as charger and the seconed one ($j=2$) with transition frequency $\omega _2=\omega _B$ will be considered as QB. It is assumed that QBs and chargers interact with a global environment and there is no direct interaction between them. QB and charger are assumed to move along the Z-axis of a cavity at a constant speed $v_i$, which in general can vary for each qubit. It is possible to write the Hamiltonian of the model as follows

9 $$\begin{aligned} H=H_{0} + f(t) H_{int}, \end{aligned}$$

Where *f*(*t*) is a dimensionless function. *f*(*t*) plays the role of an on-off switch, such that it equals to 1 for $t \in [0,\tau ]$ and zero elsewhere, and $\tau$ is the charging time of QB. It is assumed that for $t<0$ the QB and charger do not have interaction with environment. In Eq. (9), $H_{0}$ is the total free Hamiltonian which can be written as

10 $$\begin{aligned} H_{0}=\sum _{j=1}^{2} \omega _j \hat{\sigma }_+^j \hat{\sigma }_-^j + \sum _k \omega _k \hat{a}_k^{\dag } \hat{a}_k, \end{aligned}$$

where $\hat{\sigma }_{\pm }^{i}$ is the Pauli raising and lowering operators for *i*th qubit and $\omega _i$ is the transition frequency of *i*th qubit. $\hat{a}_k$ and $\hat{a}_k^{\dag }$ are the annihilation and creation operators of the *k*th mode of the cavity, respectively. The interaction Hamiltonian $H_{int}$ can be written as

11 $$\begin{aligned} H_{int}=\sum _{j=1}^{2}\sum _{k}\beta _j \hat{\sigma }_{+}^{j} g_k {\mathscr {M}}_k^j(z)\hat{a}_k+H.c. \end{aligned}$$

It should be noted that $\beta _j$ is what represents the speed of *j*th qubit inside the cavity and $g_k$ defines the coupling constant between qubits and the *k*th mode of a cavity. The motion of the qubits is limited along the *z*-axis of the cavity^40^. In the general case of two qubits moving at different speeds along the *z*-axis, the shape function of the *j*th qubit motion ${\mathscr {M}}_k^j(z)$ can be expressed as follows

12 $$\begin{aligned} {\mathscr {M}}_k^j(z)={\mathscr {M}}_k(v_j t)=\sin [\omega _k(\beta _j t - \Gamma )], \quad j=1,2, \end{aligned}$$

with $\beta _j=v_j/c,$ and $\Gamma =L/c$, where *L* is the size of the cavity and *c* is the speed of light. In the above relation, the sine term is due to the boundary conditions. The boundary conditions are such that the shape function ${\mathscr {M}}_k^j(z)$ must be non-zero at $z=0$ and zero at $z=L$. There has been a classical approach to dealing with the translational motion of the qubits ($z=v t$). It should be noted that the de Broglie wavelength $\lambda _B$ of the qubits is much smaller than the resonant transition wavelength $\lambda _0$ ($\lambda _B/\lambda _0<< 1$)^41^. So, it can be concluded that $\beta _j<<1$. The dimensionless collective coupling constant $\alpha _T$ and relative strengths $r_j$ can be written as $\alpha _T=(\alpha _1^2+\alpha _2^2)^{1/2}$ and $r_j=\alpha _j/\alpha _T$. The parameters $r_1$ and $r_2$ satisfy the $r_1^2+r_2^2=1$. The weak and strong coupling regime can be explore by changing $\alpha _T$. Let us consider the initial state which has the following form

13 $$\begin{aligned} \left| \psi _0\right\rangle =\left( c_{01}|e, g\rangle +c_{02}|g, e\rangle \right) |\textbf{0}\rangle _R, \end{aligned}$$

where $|\textbf{0}.\rangle _R$ is the multimode vacuum state. From Eq. (13), it can be seen that there exist no excitation in the modes of cavity and the qubits are in entangled state. The state of the system at time *t* can be obtained as

14 $$\begin{aligned} \begin{aligned} |\psi (t)\rangle =&c_1(t) e^{-i \omega _1 t}|e, g\rangle |\textbf{0}\rangle _R+c_2(t) e^{-i \omega _2 t}|g, e\rangle |\textbf{0}\rangle _R \\&+\sum _k c_k(t) e^{-i \omega _k t}|g, g\rangle \left| 1_k\right\rangle , \end{aligned} \end{aligned}$$

where $\left| 1_k\right\rangle$ is the state of the cavity with just one excitation in *k*th mode. By substituting Eq. (14) into the time-dependent Schr$\ddot{o}$dinger equation, the differential equations for the time-dependent probability amplitude are obtained as follows

15 $$\begin{aligned} \dot{c}_j(t)=-i \alpha _j \sum _k g_k f_k\left( z_j\right) c_k(t) e^{i \delta _k^{(j)} t}, \quad j=1,2, \end{aligned}$$

16 $$\begin{aligned} \dot{c}_k(t)=-i g_k^* \sum _{j=1}^2 \alpha _j f_k\left( z_j\right) c_j(t) e^{-i \delta _k^{(j)} t}, \end{aligned}$$

where $\delta _k^{(j)}=\omega _j-\omega _k$. It is assumed that the two qubits have same transition frequency $\omega _A=\omega _B$. So, it is concluded that $\delta _k^{(A)}=\delta _k^{(B)}$. By integrating Eq. (16) and putting its result in Eq. (15), two integro-differential equations can be obtained for $c_1(t)$ and $c_2(t)$ as follows

17 $$\begin{aligned} \begin{aligned} \dot{c}_1(t)=&-\int _0^t \sum _k\left| g_k\right| ^2 e^{i \delta _k\left( t-t^{\prime }\right) }\left( \alpha _1^2 f_k\left( v_1 t\right) f_k\left( v_1 t^{\prime }\right) c_1\left( t^{\prime }\right) \right. \\&\left. +\alpha _1 \alpha _2 f_k\left( v_1 t\right) f_k\left( v_2 t^{\prime }\right) c_2\left( t^{\prime }\right) \right) \textrm{d} t^{\prime }, \\ \dot{c}_2(t)=&-\int _0^t \sum _k\left| g_k\right| ^2 e^{i \delta _k\left( t-t^{\prime }\right) }\left( \alpha _2^2 f_k\left( v_2 t\right) f_k\left( v_2 t^{\prime }\right) c_2\left( t^{\prime }\right) \right. \\&\left. +\alpha _1 \alpha _2 f_k\left( v_2 t\right) f_k\left( v_1 t^{\prime }\right) c_1\left( t^{\prime }\right) \right) \textrm{d} t^{\prime }. \end{aligned} \end{aligned}$$

From the above relations, it can be seen that the dynamics of the system depend on the speed of the qubits. Suppose two qubits have the same velocity $\beta _1=\beta _2=\beta$. It may be useful to emphasize that at the beginning of the interaction, different positions are assumed for the qubits. The same velocity of the qubits assures us that this spatial separation between qubits does not change over time. In other words, the qubits are sufficiently separated from each other (from their initial points of interaction) that no qubit-qubit interaction will occur as time goes on. As a result of these considerations, it can be shown that Eq. (17) could be rewritten as follows

18 $$\begin{aligned} \dot{c}_1(t)=-\int _0^t F\left( t, t^{\prime }\right) \left( \alpha _1^2 c_1\left( t^{\prime }\right) +\alpha _1 \alpha _2 c_2\left( t^{\prime }\right) \right) \textrm{d} t^{\prime }, \end{aligned}$$

19 $$\begin{aligned} \dot{c}_2(t)=-\int _0^t F\left( t, t^{\prime }\right) \left( \alpha _2^2 c_2\left( t^{\prime }\right) +\alpha _1 \alpha _2 c_1\left( t^{\prime }\right) \right) \textrm{d} t^{\prime }, \end{aligned}$$

where $F\left( t, t^{\prime }\right) =\sum _k\left| g_k\right| ^2 e^{i \delta _k\left( t-t^{\prime }\right) } f_k(v t) f_k\left( v t^{\prime }\right)$ is the correlation function. Regardless of the spectral density of the environment and the speed of the qubits in the cavity, there is a fixed solution for Eqs. (18) and (19), which leads to a stable entangled state. The stable entangled state can be obtained by setting $\dot{c}_j=0$ in Eqs. (18) and (19). As a result, the long-living decoherence-free state can be obtained as follows

20 $$\begin{aligned} \left| \psi _{-}\right\rangle =r_2|e, g\rangle -r_1|g, e\rangle . \end{aligned}$$

There is no decoherence or change in the above state over time. Due to the fact that $\left| \psi _{-}\right\rangle$ does not evolve over time, the only time evolution we can observe is that of its orthogonal state

21 $$\begin{aligned} \left| \psi _{+}\right\rangle =r_1|e, g\rangle +r_2|g, e\rangle . \end{aligned}$$

The survival amplitude of the above state can be described as ${\mathscr {Q}}(t)=\left\langle \psi _{+} \mid \psi _{+}(t)\right\rangle$. It satisfies

22 $$\begin{aligned} \dot{{\mathscr {Q}}}(t)=-\alpha _T^2 \int _0^t F\left( t, t^{\prime }\right) {\mathscr {Q}}\left( t^{\prime }\right) \textrm{d} t^{\prime }. \end{aligned}$$

We assume that the two qubits interact with an environment that has a Lorantzian spectral density $J(\omega )=W^2 \lambda / \pi [(\omega -\omega _0)^2 + \lambda ^2]$, where $\lambda ^{-1}$ is the correlation time of the environment. Due to the imperfect reflection of the cavity mirrors, the cavity field spectrum exhibits a Lorentz broadening. The correlation function for qubits in such a cavity can be expressed as $f\left( t-t^{\prime }\right) =W^2 e^{-\lambda t}$^42,43^. In the limit $\lambda \rightarrow 0$, the cavity is ideal and its correlation function corresponds to constant function $f(\tau )=W^2$. In this situation, system is reduced to the Jaynes-Cummings diatomic model with vacuum Rabi frequency ${\mathscr {R}}=\alpha _T W$^44^. In the Markovian limit (small correlation times $\lambda ^{-1}$) the decay rate can be obtained as $\gamma =2 {\mathscr {R}}^2 / \lambda$. For common parameter values, the model falls between these two limits. Under these circumstances, the correlation time can be calculated as follows

23 $$\begin{aligned} \begin{aligned} F\left( t, t^{\prime }\right) =&\frac{W^2 \lambda }{\pi } \int \textrm{d} \omega \frac{\sin [\omega (\beta t-\Gamma )] \sin \left[ \omega \left( \beta t^{\prime }-\Gamma \right) \right] }{\left( \omega -\omega _0\right) ^2+\lambda ^2} \\&\times e^{-i\left( \omega -\omega _0\right) \left( t-t^{\prime }\right) }. \end{aligned} \end{aligned}$$

In continuous limit $\Gamma \rightarrow \infty$ the above integral can be solved as

24 $$\begin{aligned} F\left( t, t^{\prime }\right) =\frac{W^2}{2} e^{-\lambda \left( t-t^{\prime }\right) } \cosh \left[ \beta \bar{\lambda }\left( t-t^{\prime }\right) \right] , \end{aligned}$$

where $\bar{\lambda } \equiv \lambda +i \omega _0$. By making use of the Laplace transformation and its inverse, the solution of Eq. (22) can be obtained as^45^

25 $$\begin{aligned} \begin{aligned} {\mathscr {Q}}(t)=&\frac{\left( q_1+y_{+}\right) \left( q_1+y_{-}\right) }{\left( q_1-q_2\right) \left( q_1-q_3\right) } e^{q_1 \lambda t} \\&+\frac{\left( q_2+y_{+}\right) \left( q_2+y_{-}\right) }{\left( q_2-q_1\right) \left( q_2-q_3\right) } e^{q_2 \lambda t} \\&+\frac{\left( q_3+y_{+}\right) \left( q_3+y_{-}\right) }{\left( q_3-q_1\right) \left( q_3-q_2\right) } e^{q_3 \lambda t}, \end{aligned} \end{aligned}$$

where $q_i$’s ($i=1,2,3$) satisfy the following cubic equation

26 $$\begin{aligned} q^3+2 q^2+\left( y_{+} y_{-}+\frac{R^2}{2}\right) q+\frac{R^2}{2}=0, \end{aligned}$$

where $y_{\pm }=1\pm \beta (1+i \omega _0 /\lambda )$ and $R={\mathscr {R}}/\lambda$. Now, the time-dependent probability amplitude in Eq. (14) can be obtained as

27 $$ \begin{aligned}   c_{1} (t) =  & \left[ {r_{2}^{2}  + r_{1}^{2} {\mathcal{Q}}(t)} \right]c_{{01}}  - r_{1} r_{2} [1 - {\mathcal{Q}}(t)]c_{{02}} , \\    c_{2} (t) =  &  - r_{1} r_{2} [1 - {\mathcal{Q}}(t)]c_{{01}}  + \left[ {r_{1}^{2}  + r_{2}^{2} {\mathcal{Q}}(t)} \right]c_{{02}} . \\  \end{aligned}  $$

## Results and discussion

In this section, you will find a detailed description of all the steps involved in the charging process of the QB in the considered model. By taking the partial traces of Eq. (14) over each of the subsystems *A* and *B*, the reduced time-dependent density matrix associated with QB and charger at $t=\tau$ can be obtained as

28 $$ \begin{aligned}   \rho _{B} (\tau ) =  & |c_{2} (\tau )|^{2} |e\rangle \langle e|_{B}  + \left( {1 - |c_{2} (\tau )|^{2} } \right)|g\rangle \langle g|_{B} , \\    \rho _{A} (\tau ) =  & |c_{1} (\tau )|^{2} |e\rangle \langle e|_{A}  + \left( {1 - |c_{1} (\tau )|^{2} } \right)|g\rangle \langle g|_{A} . \\  \end{aligned}  $$

In order to study the relationship between the energy of the QB and the charger, it will be useful to study the energy changes of them. The changes in internal energy of the QB that occur within the charging process can be expressed as

29 $$\begin{aligned} \Delta E_B={\text {Tr}}\left[ H_B \rho _B(\tau )\right] -{\text {Tr}}\left[ H_B \rho _B(0)\right] , \end{aligned}$$

where $H_B=\omega _B \sigma _+^B \sigma _-^B$ is the Hamiltonian of QB, $\rho _B(\tau )$ is the state of the QB at time $\tau$. On the other hand, the internal energy changes of the charger during the charging process can be defined as

30 $$\begin{aligned} \Delta E_A={\text {Tr}}\left[ H_A \rho _A(\tau )\right] -{\text {Tr}}\left[ H_A \rho _A(0)\right] , \end{aligned}$$

where $H_A=\omega _A \sigma _+^B \sigma _-^B$ is the Hamiltonian of charger, $\rho _A(\tau )$ is the state of the charger at time $\tau$. Regarding Eq. (28), energy changes of the QB $\Delta E_B$ and charger $\Delta E_A$ can be obtained as

31 $$\begin{aligned} \Delta E_A=\omega _A(\left| c_1(\tau )\right| ^2-\left| c_1(0)\right| ^2), \quad \Delta E_B=\omega _B(\left| c_2(\tau )\right| ^2-\left| c_2(0)\right| ^2). \end{aligned}$$

$\Delta E_A$ indicates the amount of energy that the charger has lost during the charging process, and $\Delta E_B$ indicates the amount of energy stored in the QB in that time. Using Eq. (4) along with Eq. (28), one can obtain the maximum amount of work that can be extracted from a QB at the end of the charging process under the cyclic unitary operations (ergotropy) as

32 $$\begin{aligned} {\mathscr {W}}=\omega _B \left( 2 \vert c_2(\tau ) \vert ^2 -1 \right) \Theta (\vert c_2(\tau ) \vert ^2-\frac{1}{2}), \end{aligned}$$

where $\Theta (x)=\lbrace _{0 \qquad x < 0}^{x \qquad x\ge 0}$, is the Heaviside function. Here we consider the case in which $\omega _{A,B}=\omega _0$. Also we have ${\mathscr {W}}_{\max }=\omega _0$. In the following, we study the dynamical behavior of the internal energy changes of the charger and QB during the charging process and the dynamical behavior of ergotropy for two different cases.

### I. First case

We begin by assuming that the QB is completely empty, while the charger has a large amount of internal energy compared to the QB. A situation such as this occurs when the state of the composite quantum system, which includes the QB, the charger, and the environment, is as follows

33 $$\begin{aligned} |\Phi (0)\rangle =|e\rangle _A|g\rangle _B \otimes |0\rangle _{{\mathscr {E}}}, \quad (\text { set} \quad c_{01}=1, c_{02}=0 \quad \text {in Eq.}\,(13)). \end{aligned}$$

**Figure 2:**
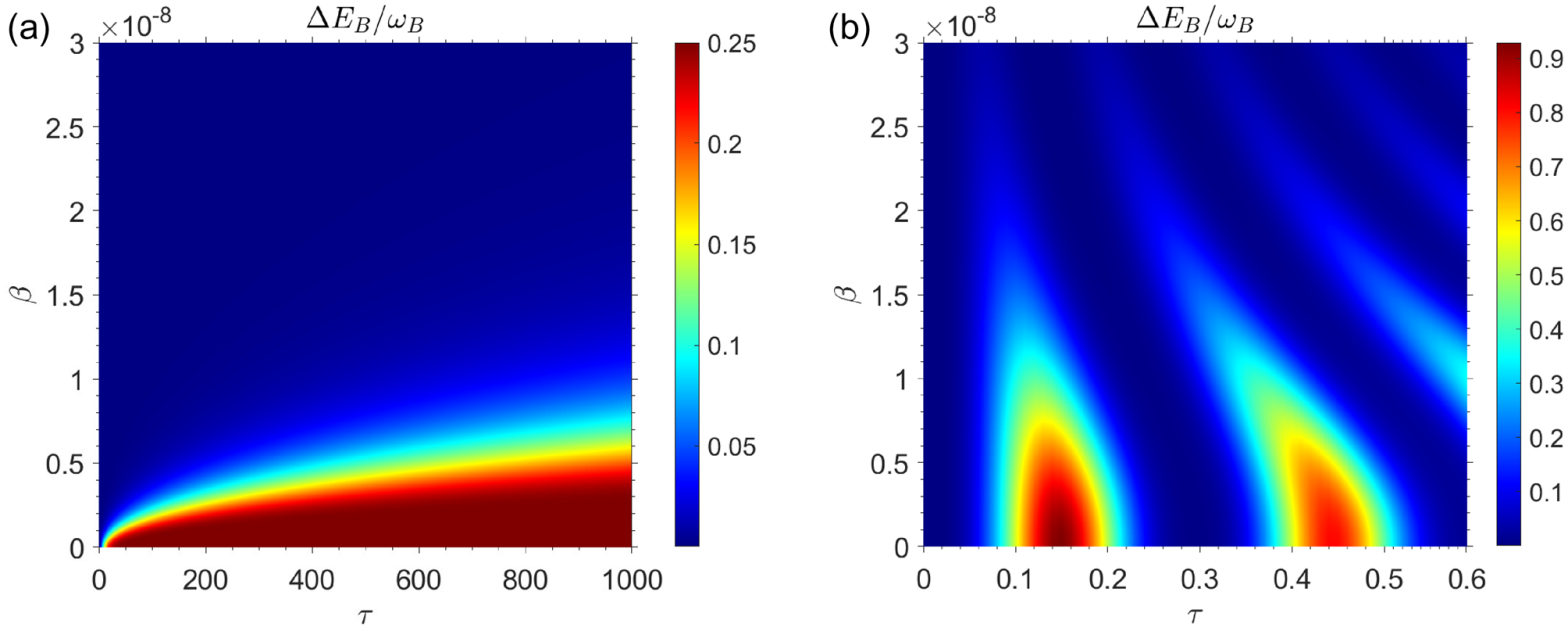
The energy changes of QB as a function of time and speed of qubits for the case in which the QB is completely empty of energy and the charger has the highest amount of energy i.e. for the initial state $|\Phi (0)\rangle =|e\rangle _A|g\rangle _B \otimes |0\rangle _{{\mathscr {E}}}$, with $\omega _0=1.5 \times 10^{9}$. (**a**) Markovian dynamics with $R=0.5$. (**b**) Non-Markovian dynamics with $R=30$.

In Fig. 2, the internal energy change of the QB is plotted in terms of the charging time $\tau$ and the speed of the moving QB $\beta$ for both the Marovian and non-Markovian regimes. According to the assumptions made previously, the initial energy of the QB is equal to zero.

**Figure 3:**
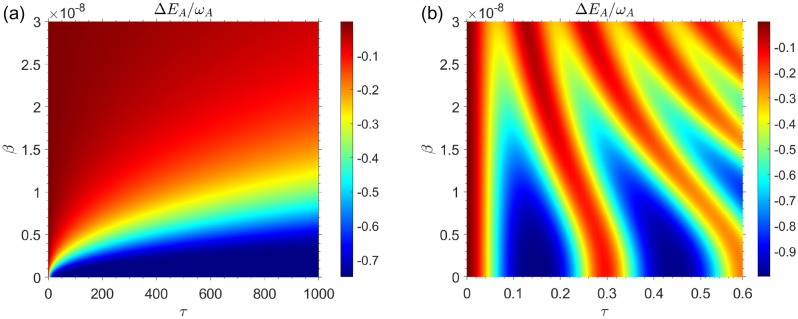
The energy changes of charger as a function of time and speed of qubits for the case in which the QB is completely empty of energy and the charger has the highest amount of energy i.e. for the initial state $|\Phi (0)\rangle =|e\rangle _A|g\rangle _B \otimes |0\rangle _{{\mathscr {E}}}$, with $\omega _0=1.5 \times 10^{9}$. (**a**) Markovian dynamics with $R=0.5$. (**b**) Non-Markovian dynamics with $R=30$.

Fig. 2a illustrates the QB’s internal energy change as a function of time $\tau$ and speed of QB inside cavity $\beta$ for the Markovian regime with $R=0.5$. In the Markovian regime $R=0.5$, it can be seen that the internal energy change of the QB decreases as the speed of the moving QB increases inside the cavity. The internal energy change of the QB $\Delta E_B$ has been plotted in Fig. 2b for non-Markovian regime with $R=30$. Similar to our observations in the Markovian regime, in the non-markovian regime the energy changes of QB decrease with increasing the speed of QB inside cavity. In comparing Fig. 2a,b, it is evident that in Markovian regime the maximum value of stored energy in the QB is $\Delta E_B^{\max } \simeq 0.25 \omega _0$ while in non-Markovian regime we have $\Delta E_B^{\max } \simeq 0.9 \omega _0$. So, it can be concluded that the amount of energy that can be stored in the QB is the greatest in the non-Markovian regime. Fig. 3, shows the internal energy changes of charger as a function of time $\tau$ and charger speed $\beta$. On the basis of the beginning assumption, it can be seen that the internal energy of charger has its maximum value at initial charging time.

**Figure 4:**
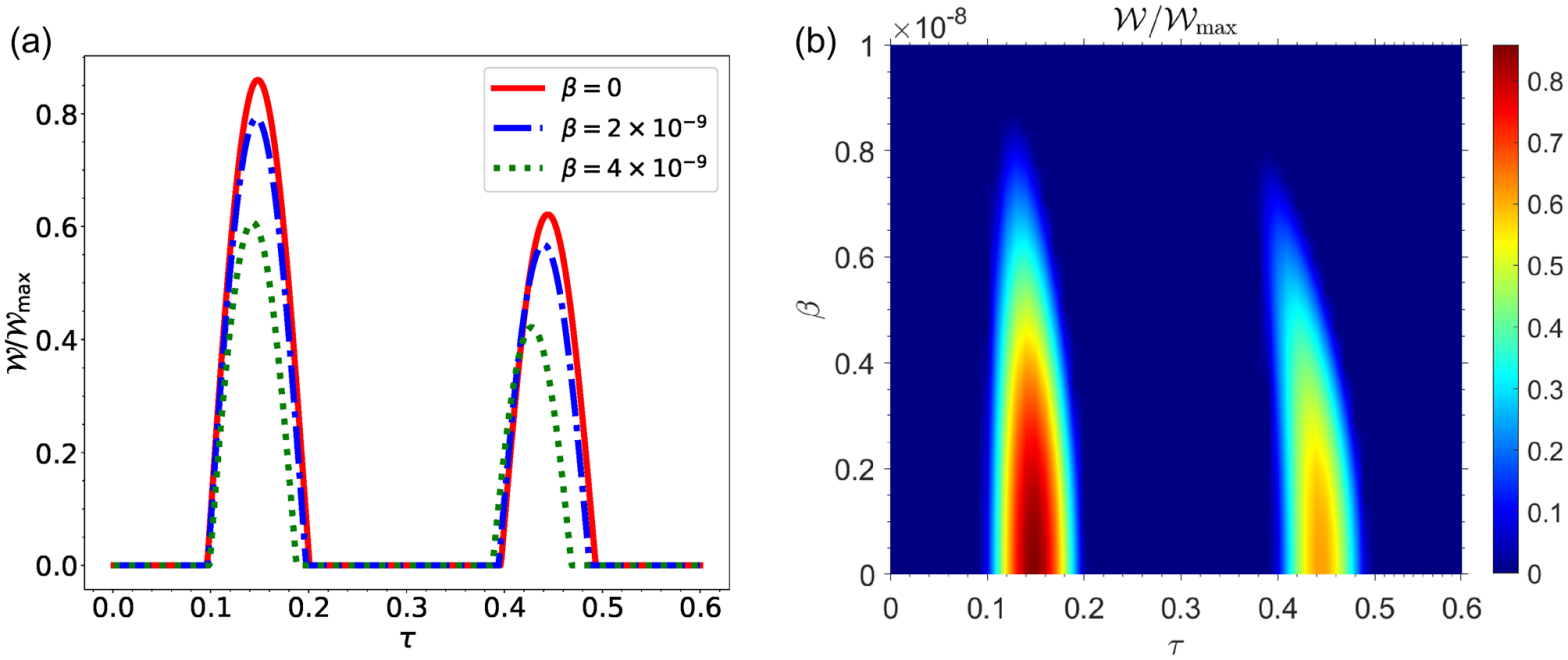
(**a**) Dynamics of ${\mathscr {W}}/{\mathscr {W}}_{max}$ as a function of time for non-Markovian regime for different values of qubit speed $\beta$, with $R=30$ and $\omega _0=1.5 \times 10^{9}$, for the initial state $|\Phi (0)\rangle =|e\rangle _A|g\rangle _B \otimes |0\rangle _{{\mathscr {E}}}$. (**b**) Density plot of ${\mathscr {W}}/{\mathscr {W}}_{max}$ as a function of time $\tau$ and qubit speed $\beta$ for non-Markovian regime, with $R=30$ and $\omega _0=1.5 \times 10^{9}$, for the initial state $|\Phi (0)\rangle =|e\rangle _A|g\rangle _B \otimes |0\rangle _{{\mathscr {E}}}$.

From Fig. 3a, it can be seen that the amount of energy used by the charger to charge the QB decreases as the charger’s speed increases in Morkovian regime. Fig. 3b shows the same results for non-Markovian regime. It can also be seen that for non-Markovian regime the amount of energy wasted in the charger to charge the QB decreases with the increase in the speed of charger. It can be seen from the Fig. 3a,b that the charger loses more energy in the non-Markovian regime than in the Markovian regime. The maximum value of energy loss in Markovian regime is $\vert \Delta E_A \vert ^{\max } \simeq 0.7 \omega _0$ while it is $\vert \Delta E_A \vert ^{\max } \simeq 0.9 \omega _0$ in non-Markovian regime. Whenever the QB is initially completely empty in the Markovian regime, no work can be extracted from the QB, and the ergotropy is equal to zero at any time (a plot of its ergotropy has not been shown here). In Fig. 4a, the ergotropy of QB in non-Markovian regime has been plotted as a function of time /*tau* for different values of QB speed $\beta$. As can be seen, as the speed $\beta$ increases, the amount of work that can be extracted from the QB decreases. In the Fig. 4b, the ergotropy has been plotted in terms of charging time $\tau$ and speed $\beta$. It can be seen that ergotropy reaches its lowest value zero as the speed of QB increases. As a general result of the first case whenever the QB is initially completely empty, it can be said that the best efficiency in the charging process will be in the non-Markovian regime. Fig. 4 shows that when the battery does not move, that is, when $\beta =0$, the maximum work can be extracted from the QB (for $\beta =0$, we have ${\mathscr {W}}=0.85 {\mathscr {W}}_{\max }$). It makes sense that a battery that is stationary will be charged more efficiently than a battery that is moving. In summary, it can be concluded that the movement of the QB in the cavity reduces the efficiency of the charging process of QB.

### II. Second case

Now we consider the case in which the initial state of the total system composed of QB, charger and environment has the following form

34 $$\begin{aligned} |\Phi (0)\rangle =\left( \alpha _{-}\left| \psi _{-}\right\rangle +\alpha _{+}\left| \psi _{+}\right\rangle \right) \otimes |0\rangle _{{\mathscr {E}}}, \end{aligned}$$

with $\alpha _{\pm }= \langle \psi _{\pm } \vert \Phi (0) \rangle$. Where $\left| \psi _{-}\right\rangle$ is a subradiant state of the Hamiltonian, which is decoherence-free, that is not affected during time evolutionas and its orthogonal state is $\left| \psi _{+}\right\rangle$. They have been introduced in Eq. (20) and Eq. (21), respectively. From a physical perspective, this state is chosen since it is less likely to be incoherent as a result of interaction with the environment. Even in a weak coupling regime (Markovian evolution), it is possible to extract work from the QB by selecting some appropriate coefficients for $\alpha _{\pm }$ and $r_{1}$, $r_2$^31^. In this case the solution for amplitude $c_1(\tau )$ and $c_2(\tau )$ can be find as

35 $$ \begin{aligned}   c_{1} (\tau ) =  & c_{2} \alpha _{ - }  + r_{1} {\mathcal{Q}}(\tau )\alpha _{ + } , \\    c_{2} (\tau ) =  &  - c_{1} \alpha _{ - }  + r_{2} {\mathcal{Q}}(\tau )\alpha _{ + } . \\  \end{aligned}  $$

**Figure 5:**
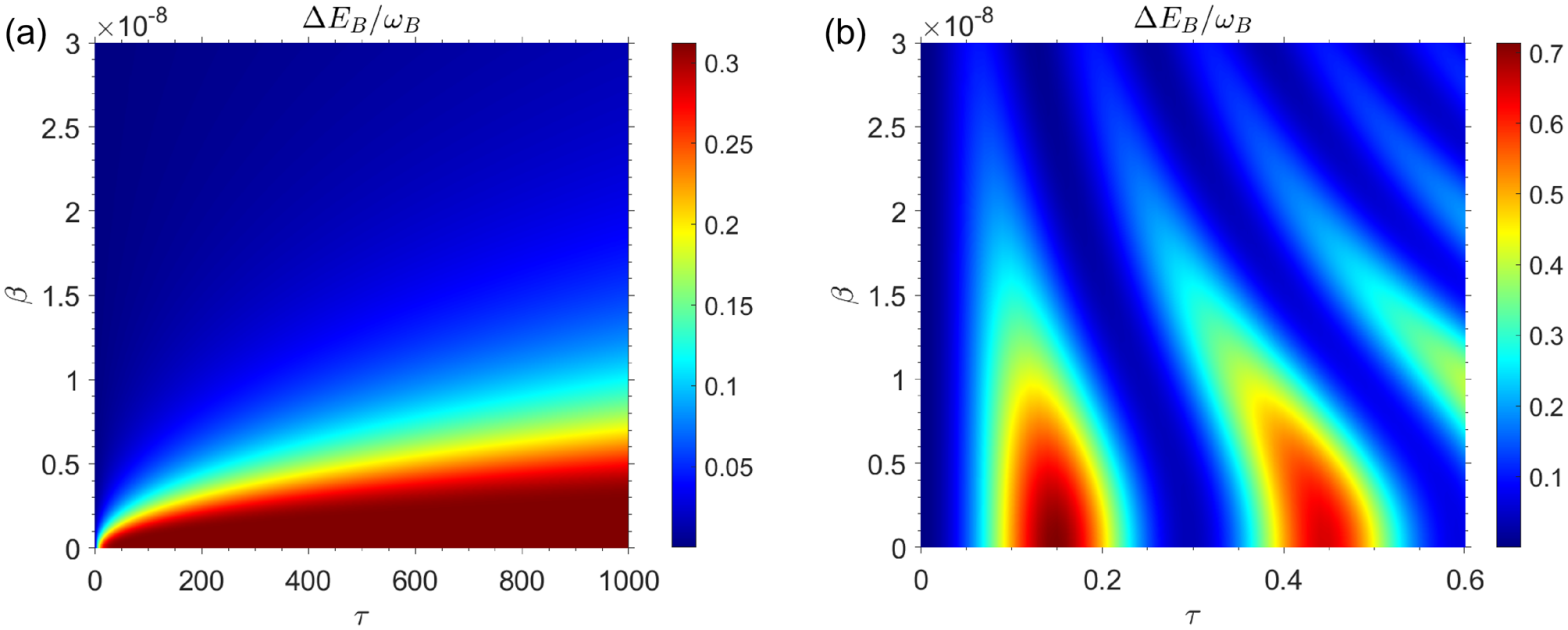
The energy changes of QB as a function of time and speed of qubits for the initial state $|\Phi (0)\rangle =\left( \alpha _{-}\left| \psi _{-}\right\rangle +\alpha _{+}\left| \psi _{+}\right\rangle \right) \otimes |0\rangle _{{\mathscr {E}}}$, with $\alpha _-=r_1=\sqrt{3}/2$ and $\omega _0=1.5 \times 10^{9}$. (**a**) Markovian dynamics with $R=0.5$. (**b**) Non-Markovian dynamics with $R=30$.

Let us consider the case where the initial state of the total system in Eq. (34), has the following coefficients $\alpha _-=r_1=\sqrt{3}/2$. In Fig. 5a, the internal energy changes of QB in Markovian regime for second case in Eq. (34) has been plotted in terms of time $\tau$ and the speed of QB $\beta$. As can be seen in this case the the maximum internal energy change of QB is $\Delta E_B^{\max } \simeq 0.3 \omega _0$. Fig. 5b, represents the internal energy changes of QB in non-Markovian regime. In non-Markovian regime the maximum value of stored energy is $\Delta E_B^{\max } \simeq 0.7 \omega _0$. From Fig. 5a,b, it can be see that the non-Markovian regimes, as compared to Markovian regimes, provide a greater amount of energy storage in QB.

**Figure 6:**
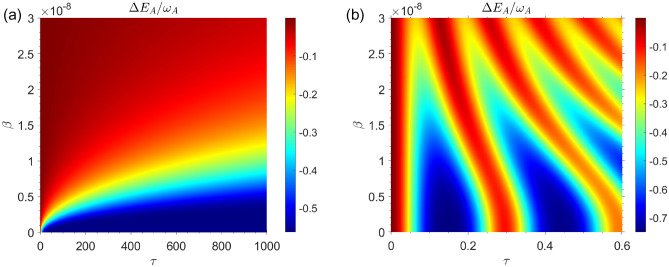
The energy changes of charger as a function of time and speed of qubits for the initial state $|\Phi (0)\rangle =\left( \alpha _{-}\left| \psi _{-}\right\rangle +\alpha _{+}\left| \psi _{+}\right\rangle \right) \otimes |0\rangle _{{\mathscr {E}}}$, with $\alpha _-=r_1=\sqrt{3}/2$ and $\omega _0=1.5 \times 10^{9}$. (**a**) Markovian dynamics with $R=0.5$. (**b**) Non-Markovian dynamics with $R=30$.

Fig. 6 shows the changes of internal energy of charger in terms of time $\tau$ and speed of charger inside environment. From Fig. 6a, it can be seen that in Morkovian regime the amount of energy wasted in the charger to charge the QB decreases with increasing the speed of the charger inside environment. Fig. 6b represents the change of charger energy in non-Markovian regime. It can also be seen that for non-Markovian regime the amount of energy wasted in the charger to charge the QB decreases with the increase in the speed charger. As the result from Fig. 6a,b, it can be seen that the highest energy loss occurs in the non-Markovian regime as compared to the Markovian regime.

**Figure 7:**
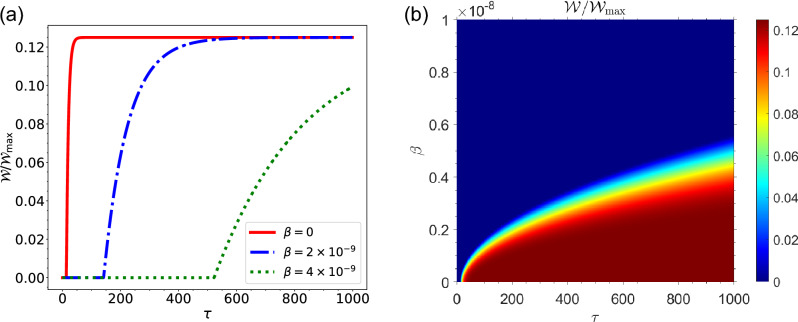
(**a**) Dynamics of ${\mathscr {W}}/{\mathscr {W}}_{max}$ as a function of time in Markovian regime for different values of qubit speed $\beta$, for the initial state $|\Phi (0)\rangle =\left( \alpha _{-}\left| \psi _{-}\right\rangle +\alpha _{+}\left| \psi _{+}\right\rangle \right) \otimes |0\rangle _{{\mathscr {E}}}$, with $\alpha _-=r_1=\sqrt{3}/2$, $R=0.5$ and $\omega _0=1.5 \times 10^{9}$. (**b**) Dynamics of ${\mathscr {W}}/{\mathscr {W}}_{max}$ as a function of time $\tau$ and qubit speed $\beta$ in Markovian regime for the initial state $|\Phi (0)\rangle =\left( \alpha _{-}\left| \psi _{-}\right\rangle +\alpha _{+}\left| \psi _{+}\right\rangle \right) \otimes |0\rangle _{{\mathscr {E}}}$, with $\alpha _-=r_1=\sqrt{3}/2$, $R=0.5$ and $\omega _0=1.5 \times 10^{9}$.

Fig. 7 shows the amount of work that can be extracted from the battery in the Markovian regime. Here, the situation is different from the previous case where the QB is initially empty and the state of total system is in product form. It should be noted that in the second case the state of the QB and charger is considered as a superposition of decoherence-free state and its orthogonal state. From Fig. 7a, it can be seen that the work can be extracted from QB, even in the Markovian regime by selecting this state with suitable coefficients for the QB and charger. As can be seen the ergotropy in Markovian regime reaches approximately to the amount of ${\mathscr {W}}=0.12 {\mathscr {W}}_{\max }$. As a result, it can be seen again that there is a decrease in the amount of work that can be extracted from the battery as the speed at which the battery is moving in the environment increases.

**Figure 8:**
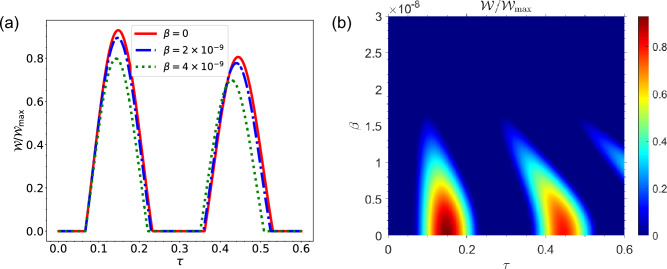
(**a**) Dynamics of ${\mathscr {W}}/{\mathscr {W}}_{max}$ as a function of time in non-Markovian regime for different values of qubit speed $\beta$, for the initial state $|\Phi (0)\rangle =\left( \alpha _{-}\left| \psi _{-}\right\rangle +\alpha _{+}\left| \psi _{+}\right\rangle \right) \otimes |0\rangle _{{\mathscr {E}}}$, with $\alpha _-=r_1=\sqrt{3}/2$, $R=30$ and $\omega _0=1.5 \times 10^{9}$. (**b**) Dynamics of ${\mathscr {W}}/{\mathscr {W}}_{max}$ as a function of time $\tau$ and qubit speed $\beta$ in non-Markovian regime for the initial state $|\Phi (0)\rangle =\left( \alpha _{-}\left| \psi _{-}\right\rangle +\alpha _{+}\left| \psi _{+}\right\rangle \right) \otimes |0\rangle _{{\mathscr {E}}}$, with $\alpha _-=r_1=\sqrt{3}/2$, $R=30$ and $\omega _0=1.5 \times 10^{9}$.

Fig. 8, represents the ergotropy in non-Markovian regime. There exist similar results as what was observed in the Markovian regime regarding the amount of work that can be extracted from the QB in the non-Markovian regime, with the difference being that the amount of work that can be extracted in the non-Markovian regime is greater than what can be extracted in the Markovian regime. From Fig. 8a,b, it can be also seen that in non-Markovian regime the amount of work that can be extracted from QB decreases with increasing the speed of QB inside environment.

## Outlook and summary

An open QB can be defined as a quantum system that is capable of storing and releasing energy under the influence of its surroundings or an external environment. This interaction with the environment is crucial as it affects the battery’s performance and efficiency. The performance and efficiency of QB can be reduced because of energy loss, decoherence, and thermalization due to the interaction with the environment. However, the environment can also be used to enhance certain aspects of the open QB. For instance, environmental coupling can assist in the initialization of the battery or enable energy transfer processes to take place in a more efficient manner. This study examined the charging process of a QB-charger that moves with a particular speed inside the common environment. In this model, wireless-like charging model has been considered and the battery and charger have no interaction with each other. In this work, two cases were considered. First, it is assumed that the battery is completely empty and that the charger has the greatest amount of energy. In the first case, when the coupling is weak, the QB can be charged while no work can be extracted from it. It is due to the fact that, in Markovian regime we do not have a back-flow of information from the environment to system^31^ and there is an amplification of decoherence effects and ergotropy is generally zero. In the strong coupling regime, the QB can be properly charged and the ergotropy is non-zero. In non Markovian regime the evolution of a QB is influenced by its environment in a way that retains memory of past interactions. This memory effect leads to the decrement of decoherence effects which makes the possibility of extracting non-zero work from the battery. In the second case, a situation was assumed in which the state of the battery and charger is considered as a superposition of decoherence-free state and its orthogonal state. In this case, the results are the same as the first case, with the difference that in the second case, due to the existence of the decoherence-free state, it is possible to extract work from the battery in Markovian regime.

Furthermore, it was observed that the movement of the QB inside the environment has an adverse effect on the charging of the QB. The speed of the QB can affect the rate of energy exchange from the environment to the QB, and thus has an effect on the performance of the charging process of the QB. It was observed that for both Markovian and non-Markovian regime the performance of QB decreases with increasing the speed of QB inside environment. It can be concluded that when the QB moves quickly, it may not have sufficient time to exchange energy with environment, which could lead to a less efficient charging process. However, when the qubit moves slowly, it may have sufficient time to exchange energy with environment, which could lead to a less efficient charging process.

## Data Availability

The datasets used and analysed during the current study available from the corresponding author on reasonable request.
